# Acceleration of Bone Regeneration Induced by a Soft‐Callus Mimetic Material

**DOI:** 10.1002/advs.202103284

**Published:** 2021-12-28

**Authors:** Alessia Longoni, Lizette Utomo, Abbie Robinson, Riccardo Levato, Antoine J. W. P. Rosenberg, Debby Gawlitta

**Affiliations:** ^1^ Department of Oral and Maxillofacial Surgery & Special Dental Care University Medical Center Utrecht Utrecht University G05.222, PO Box 85500 Utrecht 3508 GA The Netherlands; ^2^ Regenerative Medicine Center Utrecht Utrecht 3584 CT The Netherlands; ^3^ Department of Clinical Sciences Faculty of Veterinary Medicine Utrecht University Yalelaan 108 Utrecht 3584CM The Netherlands; ^4^ Department of Orthopaedics University Medical Center Utrecht Utrecht University Utrecht 3508 GA The Netherlands

**Keywords:** allogeneic, devitalization, endochondral bone tissue regeneration, mesenchymal stromal cells, orthotopic bone defect

## Abstract

Clinical implementation of endochondral bone regeneration (EBR) would benefit from the engineering of devitalized cartilaginous constructs of allogeneic origins. Nevertheless, development of effective devitalization strategies that preserves extracellular matrix (ECM) is still challenging. The aim of this study is to investigate EBR induced by devitalized, soft callus‐mimetic spheroids. To challenge the translatability of this approach, the constructs are generated using an allogeneic cell source. Neo‐bone formation is evaluated in an immunocompetent rat model, subcutaneously and in a critical size femur defect. Living spheroids are used as controls. Also, the effect of spheroid maturation towards hypertrophy is evaluated. The devitalization procedure successfully induces cell death without affecting ECM composition or bioactivity. In vivo, a larger amount of neo‐bone formation is observed for the devitalized chondrogenic group both ectopically and orthotopically. In the femur defect, accelerated bone regeneration is observed in the devitalized chondrogenic group, where defect bridging is observed 4 weeks post‐implantation. The authors' results show, for the first time, a dramatic increase in the rate of bone formation induced by devitalized soft callus‐mimetics. These findings pave the way for the development of a new generation of allogeneic, “off‐the‐shelf” products for EBR, which are suitable for the treatment of every patient.

## Graphical abstract

A devitalized soft‐callus mimetic derived from allogeneic non‐immunologically matched donor mesenchymal stem cells is engineered. In vitro, the devitalized constructs present the same characteristics of their living counterparts. In vivo the devitalized soft‐callus mimetic outperforms their living equivalent, in terms of accelerated bone regeneration and the quality of the newly formed bone.

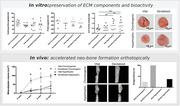

## 1 Introduction

Over the last years, an interest has grown in the use of decellularized and devitalized extracellular matrices as bioactive scaffolds for in situ tissue engineering (TE).^[^ ^1^ ^]^ These decellularization and devitalization processes entail the killing of resident cells while preserving the bioactive components of the native extracellular matrix (ECM).^[^ ^2^ ^]^ Yet, the main difference between the two methods is that with decellularization protocols, cellular debris is removed whereas this is not the case for devitalization strategies.^[^ ^2^ ^]^ The use of native or engineered decellularized and devitalized tissues as scaffolds for TE presents several advantages. The fundamental one is that the cells that are migrating into a decellularized or devitalized matrix, are surrounded by the ECM naturally present in the target tissue. Here, the biochemical cues present in the ECM promote cell attachment, migration,^[^ ^3^ ^]^ differentiation,^[^ ^1^, ^3^ ^]^ and ultimately tissue repair.^[^ ^4^ ^]^ This offers a clear benefit from a regenerative perspective compared to using less instructive scaffolds. Besides their role in enhancing the regenerative capacity of implants, ECM‐based regenerative strategies are also attractive from a translational point of view. In particular, the absence of living cells would simplify the regulations around the marketing and use of ECM‐derived products.^[^ ^5^ ^]^ Furthermore, if all immunogenic components are removed, allogeneic tissues derived from non‐immunologically matched donors could be used.^[^ ^5d^ ^]^ Finally, the implementation of ECM‐based products in clinical practice would be easier from a logistical point of view because, in contrast to living engineered tissues, as decellularized and devitalized constructs can be mass‐produced, easily stored, and used when needed.^[^ ^2^ ^]^

In line with this trend, several decellularized or devitalized options have already been explored in the orthopedic field to promote fracture healing and bone regeneration.^[^ ^4^, ^5^ ^]^ Several examples of decellularized ECM‐based scaffolds that mimic the hard callus, the late stage repair tissue of a bone fracture, have been reported in literature.^[^ ^6^ ^]^ One of the most well‐known examples is the demineralized bone matrix, an osteoinductive and osteoconductive biomaterial obtained after the removal of minerals from allogeneic bone.^[^ ^6^, ^7^ ^]^ On the contrary, a less explored area is the use of decellularized and devitalized tissues that mimic the soft, cartilaginous callus present in the early stages of fracture healing.^[^ ^6^ ^]^ Evidence supporting the feasibility of using devitalized native cartilage for endochondral bone regeneration (EBR) has been available since 1920, when Asami and Dock^[^ ^8^ ^]^ implanted boiled ear and xiphoid cartilage subcutaneously in a rabbit and observed new bone formation. Recent studies further confirmed the feasibility of using decellularized or devitalized cartilaginous templates to trigger EBR ectopically^[^ ^5^, ^9^ ^]^ and orthotopically in rodents.^[^ ^9^, ^10^ ^]^ Nevertheless, unsatisfactory results in terms of bone regeneration were observed when the decellularized or devitalized cartilaginous constructs were compared to the respective living control.^[^ ^5^, ^9^, ^10^ ^]^ Furthermore, in some cases the addition of living progenitor cells to the decellularized cartilage‐derived matrix before implantation was found to be necessary in order to observe new bone formation.^[^ ^9b,c^ ^]^ All together, these results suggest that the applied decellularization or devitalization methods lead to suboptimal regeneration, potentially caused by the loss of bioactivity of the ECM components. This led to our hypothesis that milder devitalization methods are essential to preserve the structural and biochemical integrity of the tissue's ECM. However, a downside of milder approaches is that cellular debris and DNA are retained within the implanted construct. This has shown to trigger an immune response that may hamper the regenerative process induced by non‐autologous ECM‐based scaffolds.^[^ ^5^, ^11^ ^]^ Finally, contrasting evidence is present in literature regarding the optimal timing for devitalization (e.g., after chondrogenic differentiation or hypertrophic induction) in order to maximize the conversion of the cartilage template into new bone upon implantation in vivo.^[^ ^6^, ^9^, ^12^ ^]^

**Figure 1:**
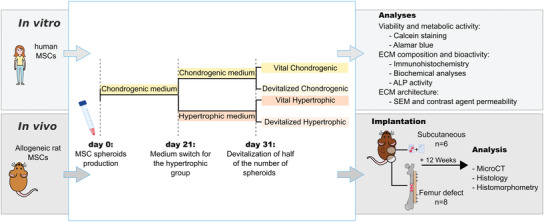
Experimental outline. For the in vitro characterization, human MSCs were embedded in collagen hydrogels and differentiated either in chondrogenic medium (31 days) or in chondrogenic + hypertrophic media (21 + 10 days). After 31 days, half of the number of the constructs of each group was devitalized. The viability of the cells and retention of ECM components were evaluated. For in vivo implantation, rat MSCs were used and an identical differentiation schedule was followed. After 31 days, spheroids were assembled in multi‐modular constructs and implanted either subcutaneously (2 spheroids per construct) or in a femur defect (8 spheroids per constructs). Carrier material control was included in the subcutaneous implantation. After 12 weeks, samples were explanted and new bone formation was evaluated as indicated.

The aim of this study was to investigate the regenerative potential of devitalized soft callus‐mimetic cartilaginous spheroids for bone TE applications. To do so, the bone regeneration induced by devitalized ECM‐based scaffolds produced after MSC chondrogenic or combined chondrogenic/hypertrophic stimulation was compared to the neo‐bone formation induced by their living counterparts. In the context of the development of an off‐the‐shelf product, all cartilaginous constructs were produced from allogeneic MSCs. The use of allogeneic cells is a key element for the clinical translation of this approach, as allogeneic MSCs could be preselected for their high chondrogenic potential, overcoming the well‐known hurdles of inter‐donor variability and unpredictability of the differentiation potential of patient‐derived cells.^[^ ^11^, ^13^ ^]^ This would ensure the access to such a treatment for every patient, irrespectively of their own cells’ potential.^[^ ^11^, ^13^, ^14^ ^]^ Moreover, the use of allogeneic cells favors the potential storage of cells, upscaling, and clinical application since larger cell numbers can be obtained from an allogeneic cell source compared to autologous, especially when pooling such cells. This is also more cost‐effective since large patient cohorts could be treated with such an allogeneic cell batch.^[^ ^13^ ^]^ To mimic a relevant clinical scenario, the regenerative potential of the engineered constructs was evaluated in a challenging and fully immunologically mismatched setting (**Figure** **1**).

## 2 Results

### 2.1 Effects of Devitalization on Human MSC‐Derived Cartilage Constructs In Vitro

**Figure 2:**
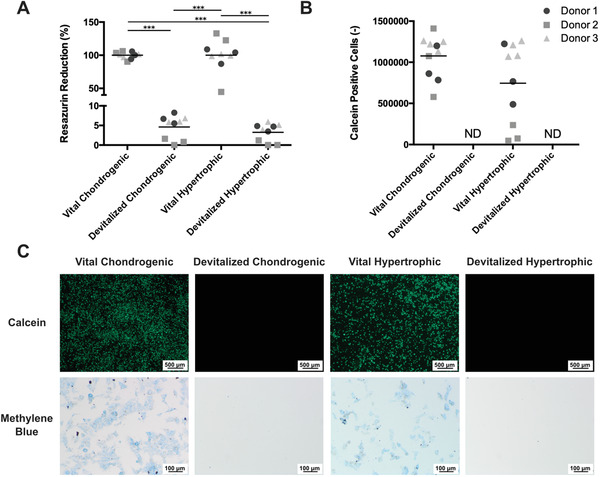
Cell viability after devitalization of engineered cartilage spheroids. A) Resazurin reduction before and after devitalization and B) the number of living cells left as detected by calcein staining (green staining). C) Calcein‐positive cells extracted from the digested constructs were stained with methylene blue after replating and culturing for 2 days. *** *p* < 0.001; ND: not detectable.

The efficacy of the devitalization procedure was investigated by evaluating resazurin reduction, which is caused by mitochondrial reductase and is a common marker for cell metabolic activity, and by calcein staining, which indicates cell viability.^[^ ^15^ ^]^ The resazurin reduction in the chondrogenic and hypertrophic samples was significantly reduced following the devitalization treatment, to 4.9 ± 2.6% and 3.7 ± 2.3% of their vital counterparts, respectively (**Figure** **2**A). After digestion of the spheroids, no calcein‐positive (living) cells were detected in the devitalized groups (Figure 2B). On the contrary, viable cells were observed for both vital groups. Furthermore, no cells attached to tissue culture plastic after the digested constructs were re‐plated, while they did for the living controls (Figure 2C). Overall, no differences in viability between the chondrogenically differentiated samples and the samples stimulated into hypertrophy were observed, although a lower number of viable cells was observed for Donor 2 in the vital hypertrophic group. After devitalization, cellular debris was still present in the devitalized construct, as confirmed by the unaffected DNA content (Figure S1, Supporting Information).

**Figure 3:**
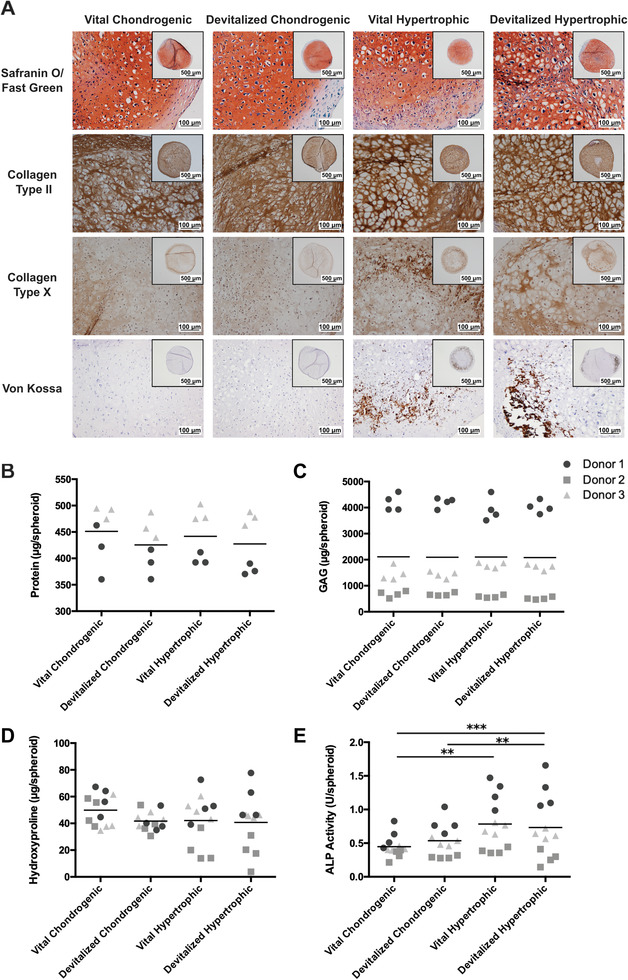
Qualitative and quantitative evaluation of the ECM preservation after devitalization of human MSC spheroids. A) Sections of spheroids from the four experimental groups were stained for GAGs (red staining) in the top row, for collagen type II and X (brown staining) and for mineralization in the bottom row (dark brown). Inserts: appearance of complete spheroids. Quantification of the B) total protein content, C) GAGs, and D) total hydroxyproline in the spheroids of the four groups before and after devitalization. E) ALP activity was quantified as an indication of the retention of the bioactivity after devitalization. ** *p* < 0.01, *** *p* < 0.001.

After assessing the effect of devitalization on cell viability, the retention of different ECM components was investigated by qualitative and quantitative analyses. Overall, ECM components were preserved after devitalization, as evaluated by histological analysis. No structural changes were apparent between vital and their respective devitalized groups. There was no evident reduction in GAGs, collagen type II, collagen type X, and calcium content in the constructs (**Figure** **3**A). These observations were supported by the quantitative assays, where no statistically significant differences were found in total protein content (Figure 3B), GAG (Figure 3C), or hydroxyproline content (Figure 3D) between the devitalized and vital samples. Moreover, ALP activity, which is more likely to be affected by the devitalization than protein content, was not reduced after the devitalization procedure (Figure 3E).

**Figure 4:**
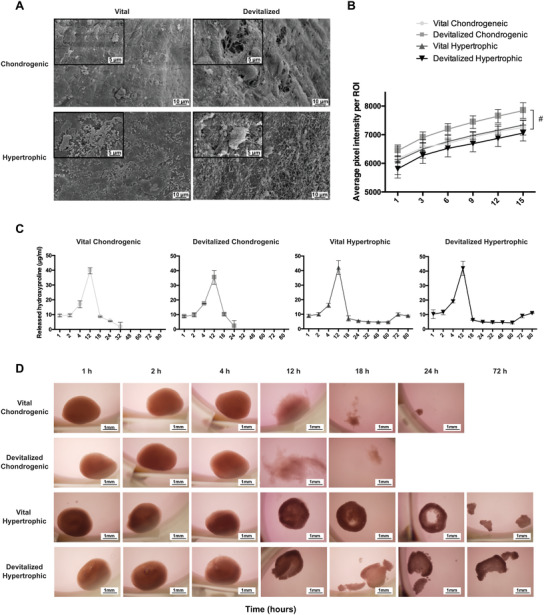
ECM porosity and degradation rate. A) SEM pictures showing the changes in surface porosity of the spheroids due to devitalization. B) Quantification of the changes in the average pixel intensity induced by the penetration of iodixanol in the selected ROI of the spheroids. C) Quantification and D) representative images of the degradation of the spheroids induced by a collagenase solution. 2 /3 of the devitalized chondrogenic spheroids were already degraded after 18 h, whereas the last one was degraded in 24 h. The majority of the vital chondrogenic spheroids (2/3 samples) was degraded by 24 h, whereas 1/3 sample was degraded by 32 h. A slower degradation rate was observed for the hypertrophic groups. #: devitalized chondrogenic group significantly different compared to devitalized hypertrophic group.

The surface roughness and porosity were evaluated by scanning electron microscopy (SEM) analysis, which highlighted an increased porosity at the surface of the chondrogenic devitalized samples, compared to the vital chondrogenic samples (**Figure** **4**A). This difference was less evident in the hypertrophic group due to the minerals present on the surface. The increased porosity in devitalized chondrogenic samples was indirectly confirmed by the highest diffusion of iodixanol (Figure 4B), a neutrally charged contrast agent, into the ROI selected at the center of the spheroids. Consistently, devitalized chondrogenic samples showed a higher degradation rate compared to other groups (Figure 4C,D). In particular, all samples from the devitalized chondrogenic group were completely degraded after a maximum of 24 h (degrading time 20 ± 3.5 h). This was 6.6 h faster than their vital counterparts (degrading time 26.6 ± 4.6 h). For the hypertrophic group, the spheroids were not fully degraded after 80 h, with no evident differences observed between the vital and devitalized groups.

### 2.2 Post‐Surgery Observations

Prior to surgery, the mean body weight of the rats was 289 ± 19 g and increased throughout the entire period, reaching 358 ± 18 g after 12 weeks. No external signs of adverse reactions (i.e., swelling or redness) at the site of implant were observed in any of the rats during the course of the experiment. One animal, of the vital chondrogenic group, died prematurely 7 weeks post‐surgery. As the cause of death was unknown, the retrieved samples were excluded from the analysis. In addition, one devitalized chondrogenic femur sample was excluded from the microCT analysis due to scattering from an inappropriately placed titanium screw. Nevertheless, this sample was still suitable for histological analysis.

### 2.3 Neo‐Bone Formation Is Induced by Devitalized Constructs when Implanted Subcutaneously

**Figure 5:**
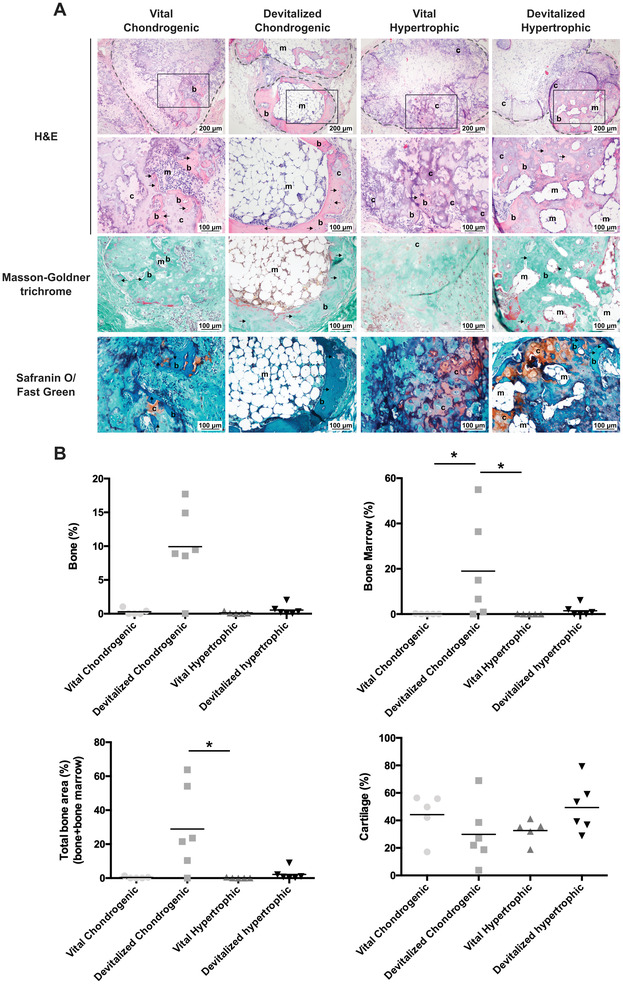
Subcutaneous endochondral bone formation 12 weeks post implantation. A) Overview of the implanted constructs stained with H&E. The grey dotted lines indicate the edges of the constructs whereas the black boxes highlight the area depicted in the higher magnification pictures in the second row. New bone formation was observed in at least a few samples of all groups (bright pink in the H&E). Furthermore, Masson–Goldner trichrome staining highlighted the presence of newly deposited osteoid (orange staining). Non‐remodeled cartilage was evident in all groups (red staining in the Safranin‐O/fast green). B) Results of the histomorphometric analysis performed 12 weeks post implantation. b: bone; c: cartilage; m: bone marrow; arrows: osteocytes. *p < 0.05.

All the collagen carrier controls were completely resorbed and could not be retrieved at the end of the study. No difference in mineralized tissue volume was observed in the microCT analysis of the subcutaneous implants, with roughly 1–3 mm^3^ mineralized tissue volume present in all groups at 12 weeks (Figure S2, Supporting Information). In contrast, differences in new bone formation were observed on histological evaluation (**Figure** **5**). In more detail, small, localized areas of bone formation were observed in the vital chondrogenic, vital hypertrophic, and devitalized hypertrophic groups (0.3 ± 0.45%, 0.12 ± 0.17%, and 0.7 ± 1.12% of the total construct area, respectively). Nevertheless, a larger amount of neo‐bone formation was observed in the devitalized chondrogenic group, with an average of 9.9 ± 6.1% of the total construct area. On average larger areas of bone marrow were also observed in this group (19 ± 22.2%) compared to the vital chondrogenic, vital hypertrophic, and devitalized hypertrophic ones. Finally, remnants of cartilage tissue were observed in all groups.

### 2.4 Devitalized Cartilage Constructs Accelerate Orthotopic Bone Formation

**Figure 6:**
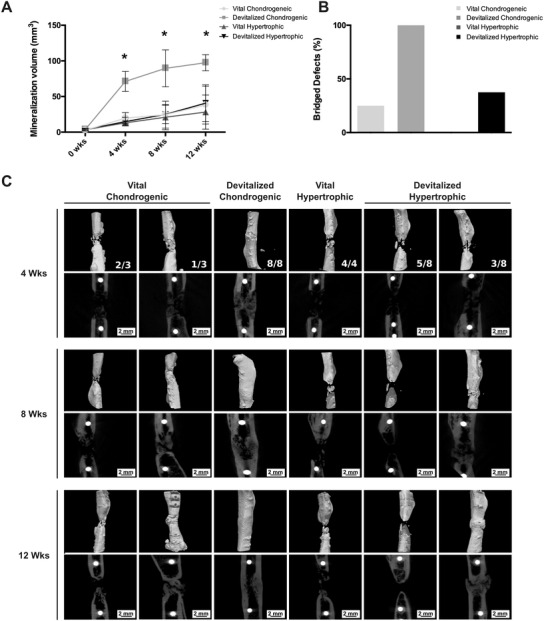
Micro‐CT‐based evaluation of bone formation in the femur defects. A) Quantification of mineralization over time for all groups. (*p < 0.05). B) Different percentages of defect bridging were observed between groups. C) 3D reconstructions and 2D images of the defects 4, 8, and 12 weeks post‐implantation. White dots: titanium screws.

**Figure 7:**
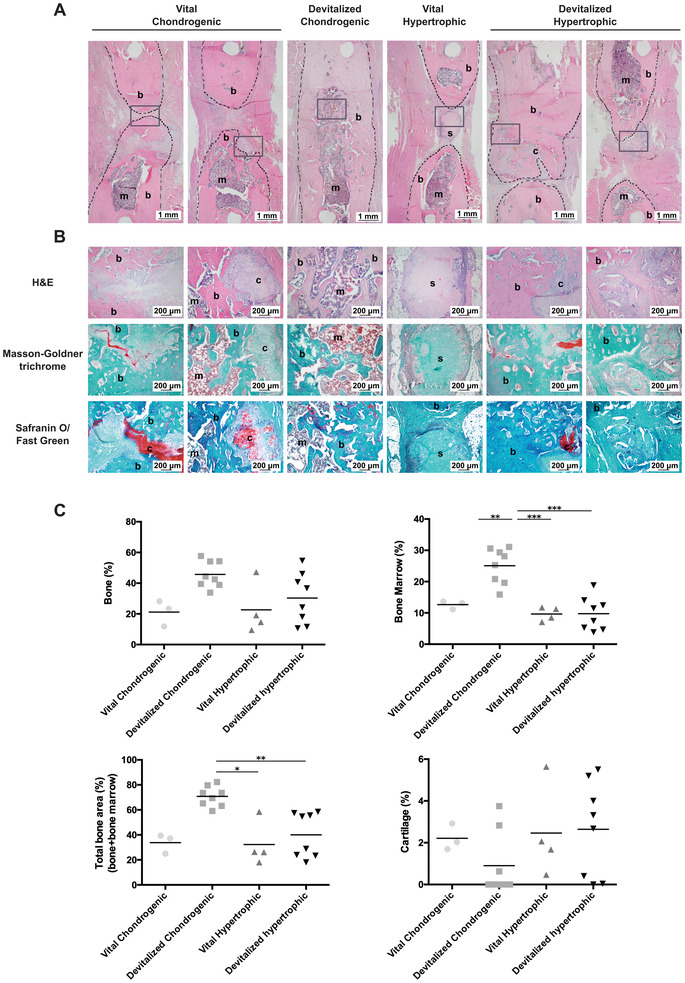
Endochondral bone formation in the femur defects 12 weeks post implantation. A) Overview of the femur defect area. The black dotted lines indicate the bone edges whereas the gray boxes highlight the magnified areas depicted in (B). B) High magnification images of H&E, Masson–Goldner trichrome, and Safranin‐O/fast green staining. C) Results of the histomorphometric performed after 12 weeks. *p < 0.05; **p < 0.01; ***p < 0.001. b: bone; c: cartilage; s: spheroid; m: bone marrow.

New mineralized bone formation was observed in all groups based on the microCT data and histological analysis (**Figures** **6** and **7**). The devitalized chondrogenic group outperformed all other groups, with a final mineralization volume of 97.4 ± 11.2 mm^3^ after 12 weeks. The mineralization volume of the devitalized hypertrophic group was 40.6 ± 25.7 mm^3^, 38 ± 26.6 mm^3^ for the vital chondrogenic group, and 28.2 ± 23.9 mm^3^ for the vital hypertrophic group (Figure 6A). In addition, a statistically significant increase in mineralization volume and complete mineralization of the defects already occurred after 4 weeks for the devitalized chondrogenic group (Figure 6A,C and Figure S3, Supporting Information). Based on microCT reconstructions, after 12 weeks, full healing was observed in all 8 samples (100%) of the chondrogenic devitalized group. On the contrary, varying results were obtained in the other groups, with full bridging in 3/8 samples of the hypertrophic devitalized group (37.5%), in 1/3 samples of the chondrogenic vital control group (33%), and in none of the samples of the vital hypertrophic control group (0%) (Figure 6B).

H&E staining showed that in the chondrogenic devitalized group, the newly formed woven bone was remodeled into lamellar bone and both the cortical and trabecular compartment of the femur was restored after 12 weeks. In just 3/8 samples, small areas characterized by the presence of hypertrophic chondrocytes (<4% of the ROI) were still observed after 12 weeks, indicating ongoing remodeling at the proximal edge of the defect (Figure 7). On the contrary, no cortical and trabecular compartments could be identified at the defect center of the other groups, not even in the radiographically fully bridged samples (Figure 6C). Traces of the non‐remodeled cartilaginous spheroids were found in the vital groups and the hypertrophic devitalized group but none in the devitalized chondrogenic group (Figure 7).

## 3 Discussion

We are the first to show accelerated bone formation by employing a devitalization strategy to engineer an allogeneic soft‐callus mimetic construct that can be applied for bone regeneration purposes. First, we demonstrated the suitability of a mild method including lyophilization to obtain devitalized cartilaginous matrices in which active biological cues are retained. Second, we showed the effectiveness of using devitalized, allogeneic, chondrogenically differentiated MSCs to trigger EBR subcutaneously and to fully regenerate critical size defects in vivo. These unique and compelling findings demarcate a significant step forward in EBR‐based strategies for bone tissue regeneration.

So far, cartilaginous template devitalization never led to bone regeneration at least comparable to the level achieved with living constructs.^[^ ^5^, ^9^ ^]^ Thus, to the best of our knowledge, this is the first time since the first report on EBR that an acceleration of the devitalized construct remodeling is presented,^[^ ^16^ ^]^ marking an important step in the light of clinical translation of this regenerative approach. The development of devitalized matrices that effectively trigger EBR enables the production of “off‐the‐shelf” constructs that could be prepared in advance from allogeneic cell sources and stored until needed.^[^ ^5a–c^ ^]^ The devitalized status brings an additional advantage to the table in the sense that it downscales the European medical agency (EMA) product classification from an advanced therapy medicinal product (ATMP) to an ECM‐based scaffold, to which less stringent regulations apply.

Our results show that devitalization does not affect the main ECM composition of samples of either the chondrogenic or the hypertrophic group. The retention of the collagenous and mineral component that we observed has been reported before using several decellularization or devitalization protocols.^[^ ^17^ ^]^ However, the preservation of GAGs is a remarkable difference compared to what is commonly reported in the literature, especially as cartilage decellularization processes often require aggressive treatments to degrade the dense ECM matrix surrounding chondrocytes.^5^, ^17^, ^18^ ^]^ Notably, the decrease of GAGs is often linked to the loss of bioactive proteins that are associated to them, such as growth factors and chemokines.^[^ ^19^ ^]^ This loss often leads to reduced bone regenerative capacity of the devitalized cartilage‐derived ECM compared to their respective vital counterparts when implanted in vivo.^[^ ^5^, ^9^ ^]^ Our data confirms the preservation of collagens and GAGs and also suggests that enzyme activity is preserved; a more in‐depth characterization of structural preservation and the presence of ECM components following devitalization will be the topic of future studies.

Preserving biochemical integrity of a cell‐derived ECM implies at the same time meager removal of cellular debris, especially in case of cartilage tissue. It has been shown that the presence of foreign DNA, mitochondria, and cellular membranes skews macrophage populations towards a pro‐inflammatory M1 phenotype, both in vitro and in vivo.^[^ ^11a,b^ ^]^ Consequently, this was reported to negatively affect tissue remodeling and the regenerative outcome in an abdominal wall defect in rats.^[^ ^11a,b^ ^]^ Nevertheless, our recent work showed that EBR could be triggered by vital, allogeneic MSC‐derived engineered cartilage, with no evidence of detrimental immune response.^[^ ^11c^ ^]^ Thus, the presence of allogeneic cellular debris was not expected to impair the bone regenerative process. Consistent with this hypothesis, in the present study, we showed that the conversion of allogeneic, devitalized spheroids into new bone tissue was not hampered by the presence of residual DNA. On the contrary, in the devitalized chondrogenic group, bridging of the femur osteotomy gap was observed in all animals already after 4 weeks and the remodeling of the newly formed tissue into mature bone, characterized by a lamellar structure and a cortical and trabecular compartment, was achieved within 12 weeks. In contrast, defect bridging was observed in only 33% of the vital chondrogenic group, which was consistent with previous results.^[^ ^11c^ ^]^ A similar trend was observed for the hypertrophic groups, where the devitalized group promoted a higher percentage of defect bridging compared to the vital hypertrophic group. The reasons why the devitalized groups outperformed their respective living counterparts could be multiple. The changes in microarchitecture induced by the devitalization process^[^ ^20^ ^]^ might have played a role. The increase in porosity and degradation rate of the samples could have favored a faster host cell infiltration, growth factor exposure, vessel ingrowth, and ultimately implant remodeling into bone tissue. Nevertheless, further studies should elucidate whether the presence of allogeneic living cells actively hampered bone formation. Specifically, the changes in environment that occurred as a consequence of in vivo implantation (e.g., oxygen tension and pH) could have triggered the secretion of stress‐response related signals (e.g., alarmins) by the implanted cells.^[^ ^21^ ^]^ This could have influenced the local recruiting of immune cells and ultimately altered the wound healing process. Thus, a comparison between the immune response triggered by the vital and devitalized spheroids, especially in the early stages post‐implantation, may lead to the identification of a specific branch of the immune response that accelerates or hinders EBR. In addition, future studies should investigate whether the newly formed bone shows adequate mechanical properties. This is clinically relevant to estimate when the newly formed bone is ready to withstand physiological load.

Several studies have shown that active enhancement of the hypertrophic phenotype in vitro preceding the devitalization^[^ ^5^, ^9^ ^]^ or decellularization^[^ ^9a^ ^]^ improves the bone regenerative outcome in vivo. In the hypertrophically stimulated constructs, the secretion of pro‐angiogenic and osteogenic growth factors, which enhance blood vessel invasion and osteogenesis in vivo, is promoted. Nevertheless, it has also been reported that ECM mineralization, as a result of the addition of *β*‐glycerophosphate in the hypertrophic medium, could have an inhibitory effect on the release of VEGF and metalloproteinase.^[^ ^22^ ^]^ This ultimately resulted in decreased pro‐angiogenic and remodeling properties of the mineralized MSC pellets in an in vitro model^[^ ^22^ ^]^ and in less bone formation in vivo.^[^ ^23^ ^]^ In addition, it must not be neglected that, even upon induction of chondrogenic differentiation, MSCs already present hallmarks of hypertrophy.^[^ ^16^ ^]^ Thus, here we evaluated whether the use of hypertrophic medium and matrix mineralization before in vivo implantation was required in order to observe the conversion of the devitalized cartilage into new bone tissue. MicroCT analysis and histological results suggest that chondrogenic differentiation of the samples—both vital and devitalized—was sufficient to achieve new bone formation in vivo ectopically and orthotopically. In particular, the chondrogenically differentiated, devitalized samples displayed enhanced bone formation at both implant locations, indicating that in our system active hypertrophy induction is not required. However, it should be noted that shorter or more prolonged hypertrophic stimulation periods could lead to other results.

All together, these results highlight the potential of a tissue engineered, allogeneic, devitalized MSC‐based cartilaginous soft callus mimic as a powerful tool to increase the clinical translatability of EBR. Our system presents several advantages over alternative strategies that apply donor/engineered tissue devitalization or decellularization, or mimic the inorganic components of bone ECM (i.e., calcium phosphates). First, as it mimics the cartilaginous soft callus, which is a temporary matrix naturally present after bone injuries, its degradation and remodeling will follow the exact events and timing of physiological fracture healing, that is, the cartilaginous template will be progressively invaded by blood vessels and remodeled by the concert action of different types of host cells such as osteoclasts and osteoblasts.^[^ ^13^, ^24^ ^]^ In other words, synchronized degradation of the engineered construct and new bone formation will occur, overcoming the challenges associated with tailoring the resorption rate of biomaterials such as calcium phosphates.^[^ ^25^ ^]^ Second, even if further analysis should be performed in order to confirm the absence of donor‐derived cells in the neo‐formed bone, in comparison to other systems, here the regenerated tissue is theoretically completely of host origin.^[^ ^9^, ^26^ ^]^ This represents an advantage compared to engineered constructs containing living chondrocytes, as they can transdifferentiate into foreign resident osteoblasts and osteocytes.^[^ ^27^ ^]^ In the presented devitalized implants, the full conversion of the implant into the patient's own bone tissue would promote a solution to problems associated with the chronic immune response elicited by slowly degrading biomaterials or living allogeneic cell implants, potentially resulting in long‐term graft rejection.^[^ ^13^, ^28^ ^]^ Third, this system will facilitate potential upscaling of the engineered constructs via modular assembly methods without encountering the problem of the formation of a necrotic core during the chondrogenic differentiation stage. The relatively small dimensions of the callus spheroids allow the optimal differentiation of the MSCs without encountering problems associated with the oxygen and nutrient diffusion limits prior to devitalization.^[^ ^9c^ ^]^ The devitalized spheroids could then serve as building blocks to create larger constructs. Afterwards, different shapes and sizes could be achieved to perfectly match the patient's defect. Nevertheless, additional studies need to be performed to confirm that upscaling to clinically relevant dimensions (from the millimeter cube to the centimeter cube range) still ensures uniform cell infiltration and tissue remodeling throughout the entire construct. Lastly, the absence of living cells increases the clinical translatability of this approach, as it potentially simplifies logistical and regulatory aspects.

## 4 Conclusion

In this study we present for the first time an allogeneic, MSC‐derived devitalized soft callus mimic that goes beyond the state of the art and outperforms its living equivalent, in terms of accelerated bone regeneration and the quality of the newly formed bone. The development of these constructs paves the way for a next generation of EBR‐based strategies, and to the potential generation of a scalable and off‐the‐shelf therapeutic product for bone restoration.

## 5 Experimental Section

### Study Design and Overview

For in vitro characterization of the effect of the devitalization process on the engineered callus‐mimetic spheroids, human MSCs were embedded in a collagen gel and chondrogenically differentiated or stimulated towards a hypertrophic state prior to devitalization. The ECM of the devitalized constructs was characterized and compared with the one of vital control samples.

For the in vivo studies, the same four groups (vital chondrogenic or hypertrophic; devitalized chondrogenic or hypertrophic) were used but with constructs derived from rat MSCs. Allogeneic rat MSCs were encapsulated in a collagen gel, differentiated, and implanted in a rat of a different strain, having a fully functional immune system. The constructs were implanted subcutaneously (*n* = 6 for each group) and in a critical size femur defect in rats. For the subcutaneous implantation, a carrier material (collagen) group was included. For the femur defect, *n* = 8 was used for the experimental conditions (devitalized chondrogenic and hypertrophic) and *n* = 4 was used for the control conditions (vital chondrogenic and hypertrophic). The regeneration induced by collagen carrier control in the orthotopic defect was already evaluated in a previous study and was proven to be very limited (≈5%).^[^ ^11c^ ^]^ For these reasons, this group was not included in the present study. A summary of previous findings for the collagen control can be found in Figure S4, Supporting Information, or in Ref. [11c]. The overall experimental outline is depicted in Figure 1.

### Isolation and Expansion of Human and Rat Bone Marrow‐Derived MSCs

Human MSCs were isolated from bone marrow aspirates of three patients (donor 1: 20‐year old, female; donor 2: 60‐year old, female; donor 3: 20‐year old, female) after informed consent, in accordance to a protocol approved by the local Medical Ethics Committee (TCBio‐08‐001‐K University Medical Center Utrecht). Ficoll‐Paque (GE Healthcare, Little Chalfont, UK) was used to separate the mononuclear fraction, which was further selected based on plastic adherence as previously described.^[^ ^29^ ^]^ Adherent cells were cultured at 37 °C under humidified conditions and 5% carbon dioxide (CO_2_) in MSC expansion medium consisting of *α*‐MEM (22 561, Invitrogen, Carlsbad, USA) supplemented with 10% heat‐inactivated fetal bovine serum (S14068S1810, Biowest, Nuaillé – France), 0.2 mm L‐ascorbic acid 2‐phosphate (A8960, Sigma‐Aldrich, St. Louis, USA), 100 U mL^−1^ penicillin with 100 mg mL^−1^ streptomycin (15 140, Invitrogen), and 1 ng mL^−1^ basic fibroblast growth factor (233‐FB; R&D Systems, Minneapolis, USA).

Rat MSCs were isolated from 4‐week old Dark Agouti rats (Envigo, Indianapolis, USA) with the approval of the Central Authority for Scientific Procedures on Animals (CCD, no. AVD1150020172465) and the animal ethical committee of the University Medical Center Utrecht. Briefly, the rats were euthanized through CO_2_ asphyxiation. After removal of the epiphysis, bone‐marrow was obtained by flushing through the diaphysis with MSC expansion medium supplemented with 0.025% ethylenediaminetetraacetic acid (EDTA). Cells were allowed to adhere in a Petri dish overnight. Afterwards, StemX Vivo medium (CCM004, R&D Systems) was used for sub‐culturing.

Both rat and human MSCs were passaged at 80% confluency until passage 4.

### Generation of MSC Callus‐Mimetic Spheroids

At passage 4, human or rat MSCs were chondrogenically differentiated. Human MSCs were used for the ECM characterization, whereas Dark Agouti rat MSCs were used for the in vivo experiments. Briefly, collagen spheroids were created by encapsulating MSCs (20 × 10^6^ mL^−1^) in 50 µL collagen type I gel droplets (4 mg mL^−1^) (354 249, Corning, New York, USA), according to the manufacturer's instructions. After gelation, the samples were cultured in serum‐free chondrogenic medium consisting of high glucose DMEM (31 966, Invitrogen) with 1% insulin‐transferrin‐selenium (ITS) + premix (354 352; Corning), 10^−7^ m dexamethasone (D8893; Sigma‐Aldrich), 0.2 mm L‐ascorbic acid 2‐phosphate (A8960, Sigma‐Aldrich), 100 U mL^−1^ penicillin, and 100 mg mL^−1^ streptomycin (15 140, Invitrogen). To differentiate human MSCs, the medium was supplement with 10 ng mL^−1^ TGF‐*β*1 (Peprotech, New Jersey, USA). For rat MSCs, also 100 ng mL^−1^ BMP‐2 (InductOS, Wyeth/Pfizer, New York, USA) was added. Medium was refreshed daily for the first 4 days and afterwards three times per week. After 21 days of chondrogenic differentiation, half of the number of spheroids was subjected to hypertrophic medium, consisting of DMEM (31 966, Invitrogen), 1% ITS + premix, 100 U mL^−1^ penicillin with 100 mg mL^−1^ streptomycin, 0.2 mm L‐ascorbic acid‐2‐phosphate, 1 nm dexamethasone, 10 mm *β*‐glycerophosphate (G9891; Sigma‐Aldrich), and 1 nm 3,3′,5‐triiodo‐L‐thyronine (T2877; Sigma‐Aldrich). Differentiation in chondrogenic or hypertrophic medium proceeded for 10 additional days till day 31.

### Devitalization Procedure of the Spheroids and Viability Analyses

At 31 days, samples were harvested and devitalized by a mild procedure including lyophilization (European Patent Application no. 20 195 800.6). To confirm devitalization, bioreduction of resazurin sodium salt (R7017; Sigma‐Aldrich) was assessed. Briefly, the vital and devitalized chondrogenic and hypertrophic constructs were incubated for 18 h at 37 °C in the dark with 500 µL of 10% resazurin sodium salt in chondrogenic medium without TGF‐*β*1. Absorbance was measured on a spectrophotometer at 570 and at 600 nm for background correction (Versamax; Molecular Devices, Sunnyvale, USA). Data are presented as percentage, considering the resazurin reduction of the vital chondrogenic and hypertrophic groups as 100%. The values obtained from empty collagen controls were subtracted.

To further confirm the absence of viable cells, constructs were digested using a 3 mg mL^−1^ collagenase type II (LS004177, Worthington; Lakewood, NJ, USA) in phosphate‐buffered saline (PBS) digest solution for a minimum of 2 h at 37 °C. The extracted cells were stained with 0.5 µg mL^−1^ Calcein‐AM (Molecular Probes, Thermo Fisher Scientific, Massachusetts, USA) for 30 min at 37 °C. Samples were excited at 495 nm and emission was registered at 515 nm (ASCENT Fluoroskan plate reader; Labsystem). For quantitative analysis, the signal was calibrated with known numbers of living MSCs to produce a standard curve. Images were acquired using an Olympus IX53 inverted fluorescence microscope. The spheroid digests were then re‐plated in a 96‐well plate and incubated with MSC expansion medium for 2 days to check for any remaining cell viability and the capacity to adhere to tissue culture plastic. Wells were washed with PBS, fixed in 10% neutral buffered formalin, and stained with methylene blue (341088‐1G, Sigma‐Aldrich) for 5 min. Images of the monolayers were taken with an Olympus IX53 inverted microscope. At least three constructs per condition for each donor were used.

### Histological Analysis of Vital and Devitalized Human MSC‐Derived Cartilage Constructs

After fixation, samples were dehydrated in a series of increasing ethanol solutions (70–100%) and cleared in xylene. Subsequently, the samples were embedded in paraffin and sliced into 5 µm thick sections (Microm HM340E; Thermo Fischer Scientific). Prior to staining, tissue sections were deparaffinized with xylene and gradually rehydrated through decreasing ethanol solutions (100–70%).

To identify cell nuclei, collagenous fibers, and glycosaminoglycans (GAGs), sections were triple stained with Weigert's hematoxylin (640 490; Klinipath BV), fast green (FN1066522; Merck), and Safranin‐O (FN1164048213; Merck). To detect mineralization, von Kossa staining was performed by incubating the sections with 1% silver nitrate (209 139, Sigma‐Aldrich) directly under a light bulb (Philips Master TL5HO 54W 830, 1 m distance), for 1 h. The samples were subsequently washed with 5% sodium thiosulfate (A17629, Alta Aesar, Haverhill, USA) and counterstained with haematoxylin.

For collagen type II (0.6 µg mL^−1^, II‐II6B3, Developmental Studies Hybridoma Bank) and collagen type X (10 µg mL^−1^, 1‐CO097‐05, clone X53, Quartett, Germany) endogenous peroxidase activity was blocked by incubating samples for 15 min with 0.3% H_2_O_2_. For collagen type II staining, antigen retrieval was done by a sequential treatment of 1 mg mL^−1^ pronase (Sigma‐Aldrich) and 10 mg mL^−1^ hyaluronidase (Sigma‐Aldrich) for 30 min each at 37 °C. For collagen type X staining, antigens were retrieved by sequential incubation with 1 mg mL^−1^ pepsin (Sigma‐Aldrich) at pH 2.0 for 2 h and 10 mg mL^−1^ hyaluronidase for 30 min, both at 37 °C. Prior to primary antibody incubation, samples were blocked with 5% BSA/PBS for 30 min at room temperature. Samples were incubated with the primary antibody overnight at 4 °C. After 30 min of incubation with the secondary BrightVision antibody (VWRKDPVM110HRP, BrightVision poly HRP‐anti‐mouse IgG, VWR, Radnor, USA), the labels were visualized by 3,3′‐diaminobenzidine oxidation. Sections were then counterstained with haematoxylin, washed, dehydrated, and mounted with Depex mounting medium. Mouse isotypes (X0931, Dako, Santa Clara, USA) were used as negative controls at the same concentration as the primary antibodies.

Images were taken with an Olympus BX51 microscope (Olympus DP73 camera, Olympus, Hamburg, Germany). Histology of empty collagen control can be found in Figure S5, Supporting Information.

### Biochemical Analysis

For total protein quantification, samples were digested with 0.5 mg mL^−1^ collagenase II for 5 h at 37 °C. Protein concentration was determined using the Pierce BCA protein assay kit (23 225, Thermo Fisher Scientific) according to manufacturer's instructions. Known concentrations of bovine serum albumin were used to create a standard curve. Absorbance was measured at 562 nm.

Samples for GAG and collagen analysis were digested overnight at 60 °C in papain digestion buffer (250 µg mL^−1^ papain, 0.2 M NaH_2_PO_4_, 0.1 EDTA and 0.01 m DL‐cysteine hydrochloride; all from Sigma‐Aldrich). The total amount of GAGs was determined using the 1,9‐dimethyl‐methylene blue (DMMB pH 3.0; Sigma‐Aldrich) assay.^[^ ^30^ ^]^ Known concentrations of shark chondroitin sulfate C (Sigma‐Aldrich) were used as standard. Absorbance values were detected at 525 and 595 nm.

To measure hydroxyproline content, 50 µL of the papain digests of all the samples were freeze‐dried overnight. Afterwards, samples were hydrolyzed by sequential incubation with 0.4 m NaOH at 108 °C and 1.4 m citric acid. Hydroxyproline contents were measured using a colorimetric method (extinction 570 nm), with chloramine‐T and dimethylaminobenzaldehyde as reagents as previously described.^[^ ^31^ ^]^ Hydroxyproline (Merck) was used as a standard.

Alkaline phosphatase (ALP) activity was measured by using the p‐nitrophenyl phosphate (pNPP) substrate system (N2765; Sigma). Different concentrations of ALP with a known activity (U per milliliter) were used as standard curve. The constructs and the standard series were incubated with the pNPP substrate at 37 °C for 8 min. Absorbance was measured at 405 nm with 655 nm as a reference wavelength.

The DNA content was quantified using a Quant‐iT Picogreen dsDNA assay (P11496, Thermo Fisher Scientific) according to the manufacturer's instructions.

Four constructs were used per condition for each donor in all analyses. Three MSC donors were used for the analysis of GAGs, collagen, ALP, and DNA. Due to inferior proliferation capacity and shortage of primary cells obtained from one donor, only two out of three MSC donors were used for the total protein quantification.

### Evaluation of the ECM Porosity and Susceptibility to Degradation

Fixed samples were dehydrated using a critical point dryer (CPD 030, Bal‐Tec) for SEM. After gold sputtering (JEOL, JFC‐1300, JEOL Ltd, Tokyo, Japan), samples were imaged using a SEM (JEOL JSM‐5600, JEOL Ltd).

For the degradation study, samples were incubated with 10 U mL^−1^ collagenase II (Worthington) in plain DMEM at 37 °C. Medium was collected and completely replaced after 1, 2, 4, 12, 18, 24, 36, 48, 60, 72, and 80 h. The collected medium was processed as described above to measure hydroxyproline content.

To indirectly measure the porosity of the constructs, samples were immobilized at the bottom of a custom‐made mold of 3% agarose gel. The top of the spheroid was exposed to Visipaque solution (iodixanol, GE Healthcare) and microCT images (Quantum FX; PerkinElmer, Waltham, USA) were taken at different time points (20 µm resolution, voltage 90 kV, current 180 mA, field of view = 10 mm). For each spheroid, the diameter was measured and a ROI of 0.1 × 1.8 mm was selected at the center of the spheroid. The changes in average pixel intensity within the ROI due to the inward diffusion of the contrast agent were monitored over time using the image processing software Image‐J (Java, Redwood Shores, USA).

One MSC donor was used to evaluate construct porosity and susceptibility to degradation. A triplicate was used for the quantitative measures whereas one sample per group was used for qualitative images.

### Construct Preparation for In Vivo Implantation

Chondrogenic differentiation and metabolic activity of the Dark Agouti MSCs was verified prior to in vivo implantation (Figures S6 and S7, Supporting Information). For subcutaneous implantation, two chondrogenic spheroids per group (vital chondrogenic and hypertrophic; and devitalized chondrogenic and hypertrophic) were embedded in collagen (4 mg mL^−1^) and cast in custom‐made square cuboid molds (3 mm x 3 mm x 2 mm). Gelation was allowed for 45 min at 37 °C according to manufacturer's instructions. Empty collagen controls were included as controls. For the orthotopic defects, eight chondrogenic spheroids were encapsulated in collagen gel in 3.5 mm x 3.5 mm x 6 mm custom‐made molds, as described above. The constructs were prepared the day before implantation and incubated overnight in a chondrogenic differentiation medium without TGF‐*β*1 and BMP‐2.

### Animal Experiment and Surgical Procedures

The animal experiments were performed with the approval of the Central Authority for Scientific Procedures on Animals (Dutch national CCD) and of the local animal welfare body (2465‐2‐01) in accordance with the ARRIVE guidelines for animal experimentation.^[^ ^32^ ^]^ The power analyses used to determine the number of samples required per group are presented in the Supporting Information. Twenty‐four male Brown Norway rats of 11 weeks old (Envigo) were randomly housed in pairs at the Central Laboratory Animal Research Facility of the Utrecht University. Animals received standard food pellets and water ad libitum, under climate‐controlled conditions (21 °C; 12 h light/12 h darkness). After 7 days of acclimatization, subcutaneous pockets were created under general anesthesia from 5 mm dorsal incisions and blunt dissection as previously described^[^ ^9b^ ^]^ (1‐3.5% isoflurane in oxygen, AST Farma, Oudewater, the Netherlands). In each pocket, one construct of either group (collagen control, vital chondrogenic, vital hypertrophic, devitalized chondrogenic, or devitalized hypertrophic) was implanted (*n* = 6 per group). The skin was closed transcutaneously with Vicryl Rapide 4‐0 sutures (VR 2297; Ethicon). Each animal received a maximum of 2 subcutaneous pockets. For implantation of the construct in a femur defect, a 6‐mm critical‐size segmental bone defect was created as previously described^[^ ^33^ ^]^ (*n* = 8 for the devitalized chondrogenic and hypertrophic experimental groups and *n* = 4 for the vital chondrogenic and hypertrophic controls). Briefly, the right hind leg was shaved and carefully disinfected. A lateral skin incision was made and soft tissue was dissected in order to expose the right femur. After the periosteum removal, three proximal and three distal screws were used to stabilize a 23 × 3 × 2 mm polyether ether ketone (PEEK) plate to the femur in the anterolateral plane. After fixation, a saw guide and a wire saw (RISystem, Davos, Switzerland) were used to remove a 6‐mm cortical bone segment. The collagen constructs were press‐fit into the defect and a single dose of antibiotic (Duplocillin LA, 22.000 IE/kg, MSD Animal Health, Boxmeer, the Netherlands) was locally injected intramuscularly. The fascia and skin were sutured in layers using resorbable Vicryl Rapide 4‐0 sutures (Ethicon). Subcutaneous injection of pain medication (buprenorphine, 0.05 mg kg^−1^ bodyweight, AST Farma, Oudewater, the Netherlands) was given pre‐operatively and twice a day for the following 3 days. When devitalized constructs were implanted in the orthotopic defect, the rats also received only devitalized constructs subcutaneously. Rats were euthanized 12 weeks after surgery with an overdose of barbiturates (phenobarbital; 200 mg kg^−1^ body weight, TEVA Pharma, Haarlem, the Netherlands). The femora and the subcutaneous implants were retrieved and processed for histological analysis and micro‐computed tomography (microCT) scanning.

### MicroCT Scanning

Mineralization in the orthotopic defect area was assessed at 0, 4, 8, and 12 weeks after surgery. While under general anesthesia, the hind leg of the rat was fixed to a custom‐made support to allow scanning of the femur with a microCT imaging system (Quantum FX). Three minutes of scan time was required per leg for an isotropic voxel size of 42 µm resolution (voltage 90 kV, current 180 mA, field of view = 21 mm). All scans were oriented in the same fashion using the ImageJ plugin Reorient3 TP (Image‐J 2.0.0; Java, Redwood Shores, CA, USA). A volume of interest (VOI) of 6.3 × 5 × 5 mm^3^ was selected. After euthanasia, subcutaneous implants were also analyzed. Three minutes of scan time was required per subcutaneous implant for an isotropic voxel size of 20 µm resolution (voltage = 90 kV, current = 180 mA, field of view = 10 mm). After segmentation with a global threshold, the mineralized volumes (MV) for both the subcutaneous and femur implants were measured in millimeter cube using the image processing software plugin BoneJ^[^ ^34^ ^]^ (Image). 3D reconstructions of the femur defect were based on the microCT data and created using ParaView (ParaView 5.3.0, Kitware Inc., USA).

### Histological Analysis of the In Vivo Samples

All specimens were fixed in a 10% neutral buffered formalin solution for 1 week and thereafter decalcified for 6 weeks in a 10% EDTA‐phosphate buffered saline solution (pH 7.4). After decalcification, samples were additionally fixed for 2 days, dehydrated in a Leica ASP300S tissue processor in graded ethanol solutions (70–100%), cleared in xylene, embedded in paraffin and sliced into 5 µm thick sections (Microm). Before staining, samples were deparaffinized with xylene and gradually rehydrated through decreasing ethanol solutions (100–70%). New bone formation was evaluated using H&E, Masson–Goldner trichrome staining and Safranin‐O/fast green staining.

Histomorphometric analysis was performed for both the subcutaneous and orthotopic samples after H&E staining. Briefly, an overview of the whole sample was made by merging images into a panoramic image in Adobe Photoshop C6. For the subcutaneous implants, bone formation throughout the entire construct area was quantified. For the orthotopic implants, a region of interest (ROI) of 6.5 × 5 mm^2^ was selected in the center of the defect. The titanium screw holes present on each side of the defect were used as reference points in order to ensure an equivalent positioning of ROI in all samples. Three different areas were manually selected for each ROI: bone, hypertrophic cartilage, and bone marrow. The number of pixels for each area was quantified via the function “recording measurement” and expressed as a percentage of the total construct area for the ectopic implants and of the ROI area in the orthotopic ones. The sections were scored independently by two scientists and the results are presented as an average.

### Statistics

A randomized block design with Bonferroni's post hoc correction was applied for the in vitro data to accommodate donor variation, including viability and biochemical analyses (protein, GAG, hydroxyproline and DNA content and ALP activity). A linear mixed model followed by a Bonferroni's post hoc correction was used to compare mineralization in the femur defect over time and to evaluate statistical differences in the Visipaque diffusion test (IBM SPSS 22.0, New York, USA). For the histomorphometric measures, when data were normally distributed, a one‐way ANOVA test was performed, followed by Tukey post‐hoc test (GraphPad Prism 6, San Diego, CA, USA). When the condition of normality was not satisfied, a Kruskal–Wallis test, followed by a Dunn's post hoc test was performed. Differences were considered to be statistically significant for *p* < 0.05.

## Conflict of Interest

The authors declare no conflict of interest.

## Supporting information

Supporting Information

## Data Availability

The data that support the findings of this study are available from the corresponding author upon reasonable request.
