# Supplementary material for: Acceleration of Bone Regeneration Induced by a Soft‐Callus Mimetic Material
Source: Adv Sci (Weinh). 2021 Dec 28;9(6):2103284. doi: 10.1002/advs.202103284 (PMC8867155; doi:10.1002/advs.202103284)
Supplement: Supplementary file 1 — Supporting Information [file ADVS-9-2103284-s001.pdf]

ADVANCED SCIENCE
Open Access

Supporting Information

for *Adv. Sci.,* DOI: 10.1002/advs.202103284

Acceleration of Bone Regeneration Induced by a Soft-Callus Mimetic Material

*Alessia Longoni, Lizette Utomo, Abbie Robinson, Riccardo Levato, Antoine JWP Rosenberg, Debby Gawlitta**

Supporting Information

Acceleration of Bone Regeneration Induced by a Soft-Callus Mimetic Material

*Alessia Longoni, Lizette Utomo, Abbie Robinson, Riccardo Levato, Antoine JWP Rosenberg, Debby Gawlitta**

Supporting Materials and Methods

To determine the sample size for the subcutaneous and femur defect, power analysis has been performed using an online tool (http://homepage.stat.uiowa.edu/~rlenth/Power/). For the orthotopic defect, the calculation was based on the data collected by van der Stok et al. (1) and from a pilot experiment previously performed in our laboratory (data not published). A one-way ANOVA with Tukey/HSD post hoc correction has been performed. For the experimental conditions (devitalized groups) a sample size of 8 implants per condition was defined to be required to detect a contrast of 30% with a power of 74%, a standard deviation of 0.17 and α=0.05. For the controls, a reduced n was selected because for the chondrogenic group data regarding bone formation were already available from a previous study (2). For the subcutaneous model, the calculation was based on the data collected by Visser et al. (3) and Gawlitta et al. (4). A one-way ANOVA with Tukey/HSD post hoc correction has been performed. A sample size of 6 implants per condition was defined to be required to detect a contrast of 25% with a power of 85%, a standard deviation of 0.13 and α=0.05.

Supporting Figures

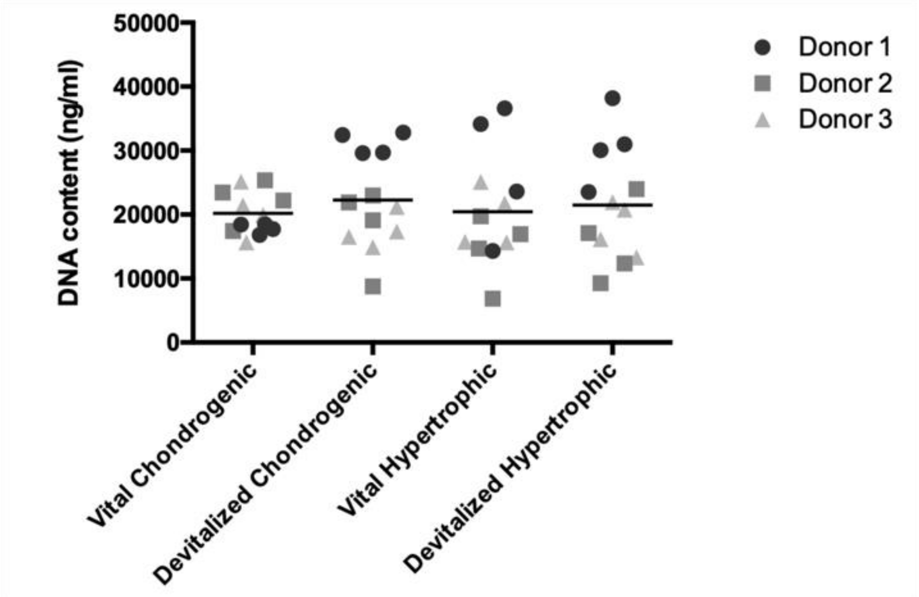

Figure S1. DNA content of the human MSC-derived spheroids after 31 day of culture. DNA content was not significantly affected by the culture medium (chondrogenic or hypertrophic) or the devitalization procedure.

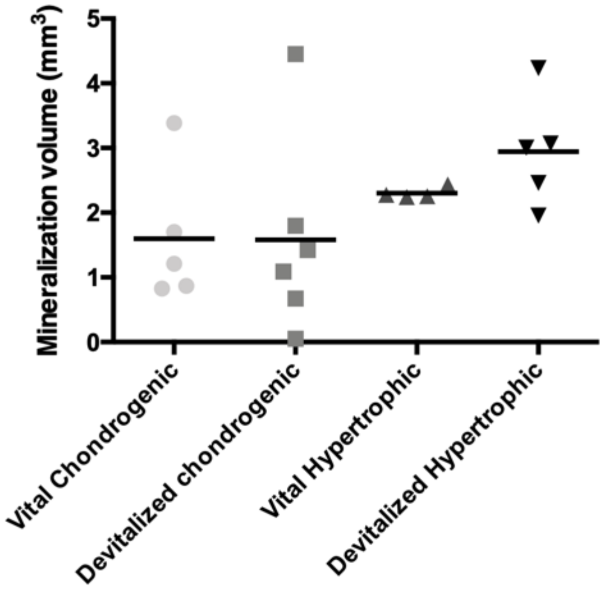

Figure S2. Mineralization of the subcutaneous implants from the four groups after 12 weeks.

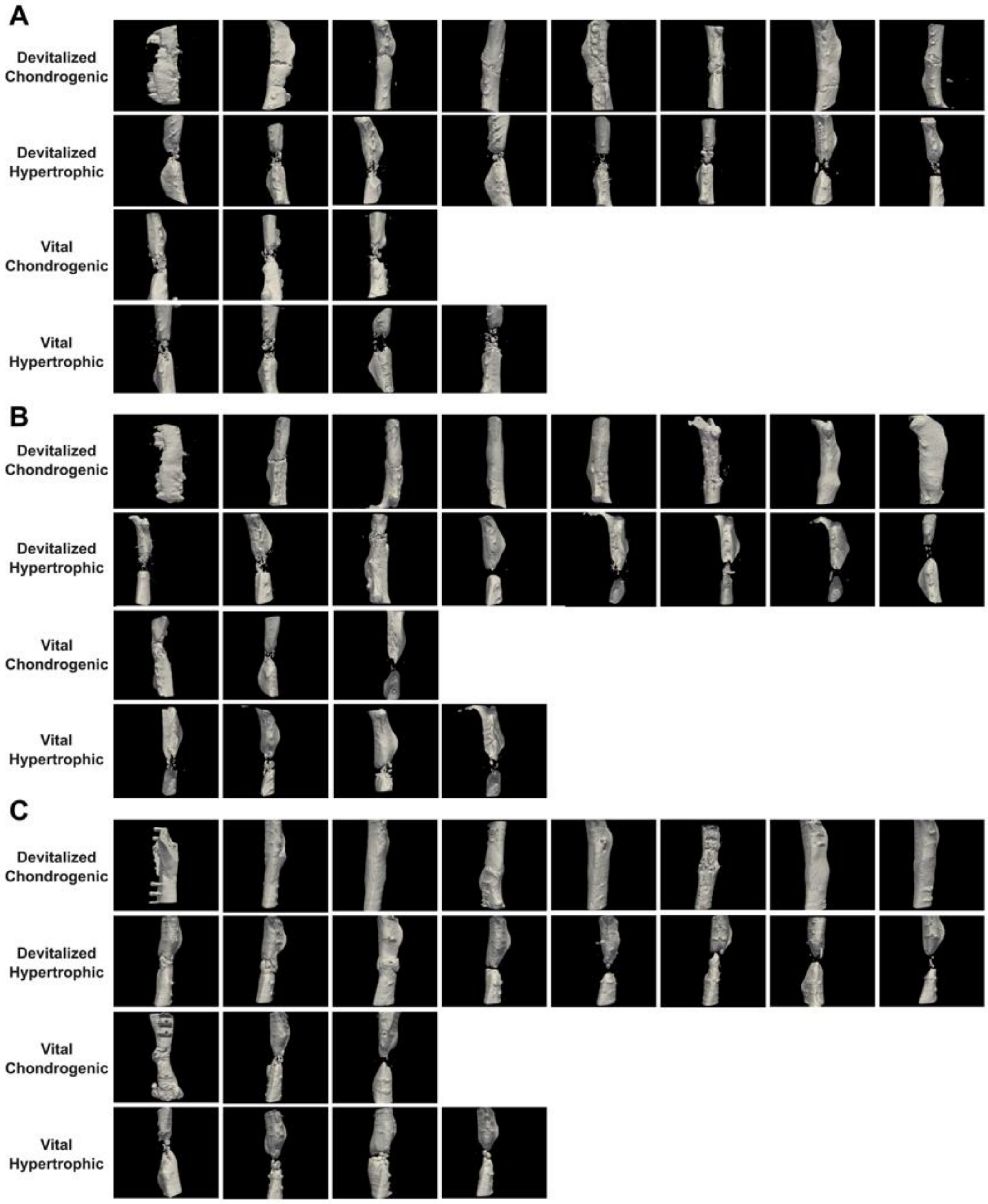

Figure S3. 3D reconstructions of the femur defects. (A) 3D reconstructions after 4 weeks, (B) 8 weeks and (C) 12 weeks.

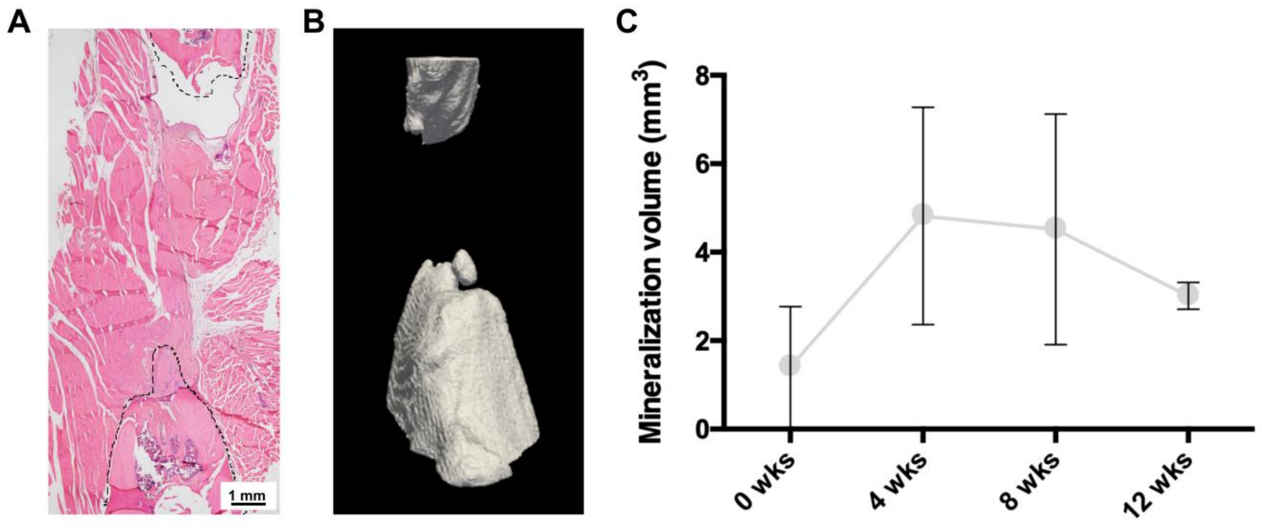

Figure S4. Overview of the regeneration induced by the collagen carrier control in the orthotopic defect. (A) H&E staining of the defect area highlights the presence of muscles and fat tissue in the center of the defect 12 weeks after surgery. The black dotted lines indicate the bone edges. (B) 3D reconstructions of the femur defects confirm that no mineralization occurred in the central area of the defect after 12 weeks of implantation. (C) The volume of mineralized tissue was observed over time. As a reference, fully bridged samples presented an average mineralized volume of 97.4 ± 11.2 mm^3. Adapted from (2), under the terms of the Creative Commons CC-BY 4.0 License (https://creativecommons.org/licenses/by/4.0/).

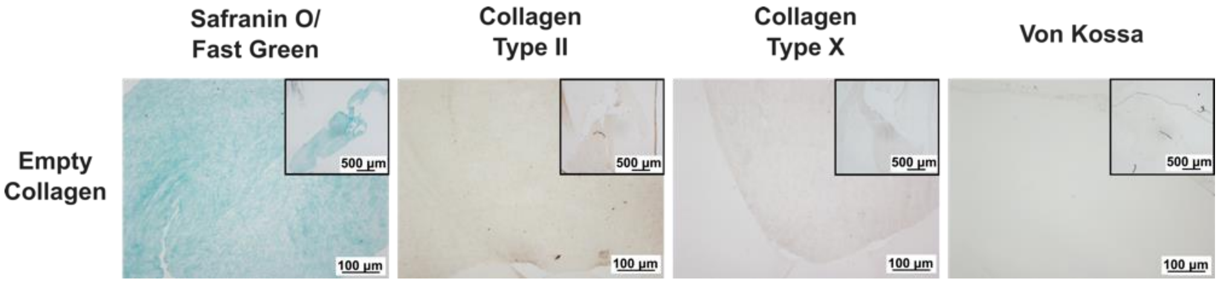

Figure S5. Histology of the empty collagen control samples after 31 days of culture. Representative images show no presence of GAGs, collagen type II or X, or mineralization. Inserts: sample overview.

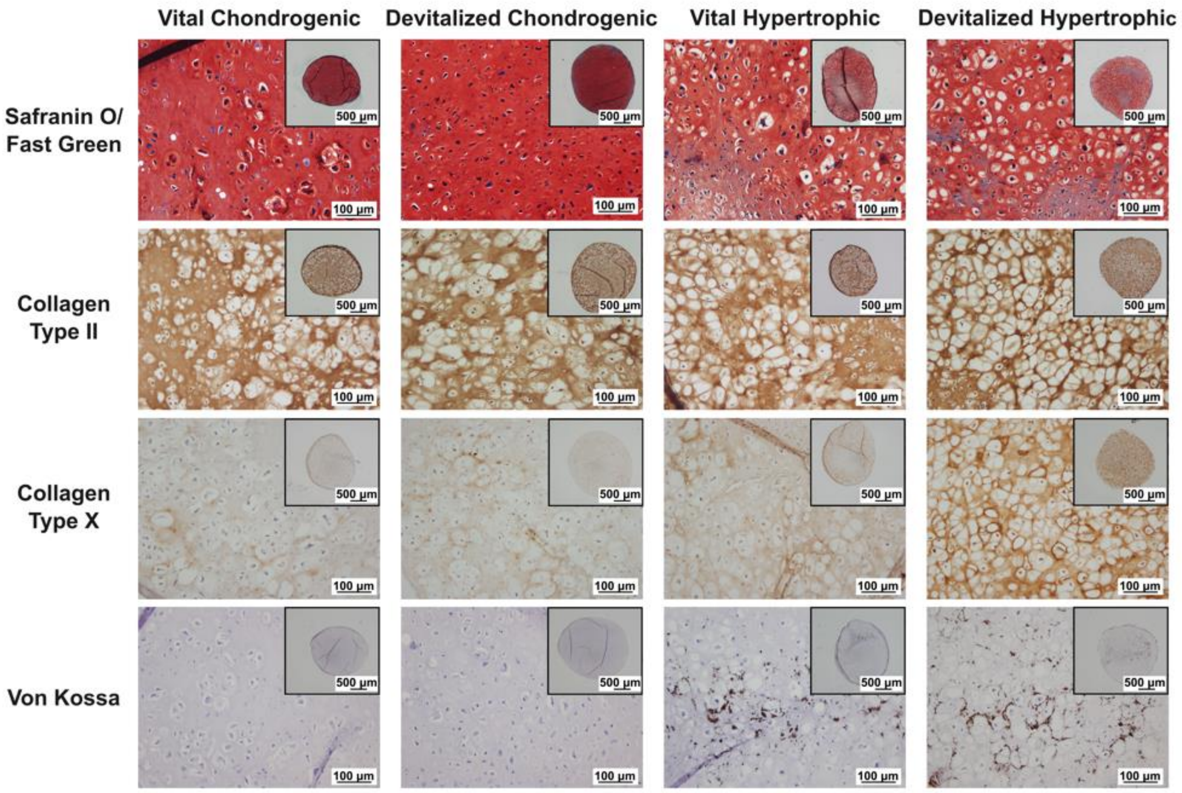

Figure S6. Characterization of the rat MSC spheroids before *in vivo* implantation. Chondrogenic differentiation was confirmed by the presence of GAGs (red staining) and collagen type II (brown staining) in all groups. Increased deposition of collagen type X and mineralization were observed in

the vital and devital hypertrophic groups. No difference in ECM components could be detected after the devitalization procedure. Inserts: spheroid overview.

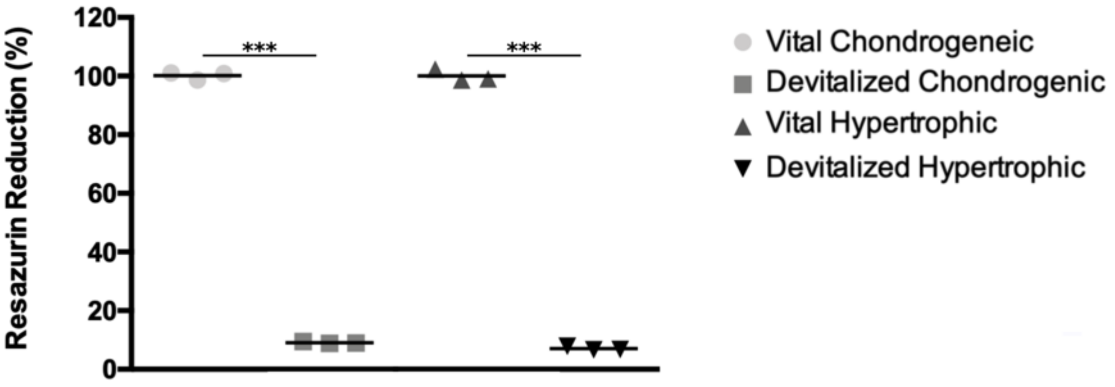

Figure S7. Metabolic activity of the rat MSC spheroids before *in vivo* implantation. Similar to the results obtained with the human MSCs, the metabolic activity of rat MSCs was reduced to below 10% for both devital groups. ***p<0.001.

References

1. van der Stok J, Koolen MK, Jahr H, Kops N, Waarsing JH, Weinans H, et al. Chondrogenically differentiated mesenchymal stromal cell pellets stimulate endochondral bone regeneration in critical-sized bone defects. Eur Cell Mater. 2014;27:137-48; discussion 48.
2. Longoni A, Pennings I, Cuenca Lopera M, van Rijen MHP, Peperzak V, Rosenberg A, et al. Endochondral Bone Regeneration by Non-autologous Mesenchymal Stem Cells. Frontiers in bioengineering and biotechnology. 2020;8:651.
3. Visser J, Gawlitta D, Benders KE, Toma SM, Pouran B, van Weeren PR, et al. Endochondral bone formation in gelatin methacrylamide hydrogel with embedded cartilage-derived matrix particles. Biomaterials. 2015;37:174-82.
4. Gawlitta D, Benders KE, Visser J, van der Sar AS, Kempen DH, Theyse LF, et al. Decellularized cartilage-derived matrix as substrate for endochondral bone regeneration. Tissue engineering Part A. 2015;21(3-4):694-703.
